# Personalizing dental screening and prevention protocols in dentulous patients with oropharyngeal cancer undergoing radiotherapy: A retrospective cohort study

**DOI:** 10.1016/j.ctro.2024.100759

**Published:** 2024-03-07

**Authors:** Denzel Chin, Hetty Mast, Gerda M. Verduijn, Michelle Möring, Steven F. Petit, Frederik R. Rozema, Eppo B. Wolvius, Brend P. Jonker, Wilma D. Heemsbergen

**Affiliations:** a Department of Radiotherapy, Erasmus MC Cancer Institute, University Medical Center Rotterdam, Dr. Molewaterplein 40, 3015 GD Rotterdam, The Netherlands; b Department of Oral and Maxillofacial Surgery, Erasmus MC Cancer Institute, University Medical Center Rotterdam, Dr. Molewaterplein 40, 3015 GD Rotterdam, The Netherlands; c Academic Centre for Dentistry Amsterdam, Gustav Mahlerlaan 3004, 1081 LA Amsterdam, The Netherlands; d Department of Oral and Maxillofacial Surgery, Amsterdam UMC, University of Amsterdam, De Boelelaan 1118, 1081 HZ Amsterdam, The Netherlands

**Keywords:** Head and neck cancer, Radiotherapy, Tooth extraction, Radiation caries, Periodontal disease

## Abstract

**Objectives:**

Patients with head and neck cancer are routinely screened for dental foci prior to radiotherapy (RT) to prevent post- RT tooth extractions associated with an increased risk of osteoradionecrosis. We evaluated the risk factors for post-RT tooth extraction to personalise dental screening and prevention protocols prior to RT.

**Materials and methods:**

This retrospective cohort study included dentulous patients diagnosed with oropharyngeal cancer who had undergone radiation therapy at doses 60–70 Gy and achieved a disease-free survival of ≥ 1 year (N = 174). Risk factors were assessed using Cox regression models.

**Results:**

The cumulative incidence of post-RT tooth extraction was 30.7 % at 5 years. Main indications for extraction (n = 62) were radiation caries (n = 20) and periodontal disease (n = 27). Risk factors associated (p < 0.05) with radiation caries-related extractions included active smoking, alcohol abuse, poor oral hygiene, parotid gland irradiation, and mandibular irradiation. A high-dose volume in the mandible was associated with periodontal disease events.

**Conclusion:**

Post-RT extractions due to radiation caries were influenced by lifestyle factors and RT dose in the mandible and parotid glands. Periodontal disease-related extractions were primarily associated with the mandibular dose. During dental screening these post-RT risk factors should be taken into account to prevent osteoradionecrosis.

## Highlights

• Post-radiotherapy teeth extractions within 5 years occurred in 31 %.
• Main indications radiation caries and periodontal disease.
• Dose factors and life-style factors related to radiation caries extractions.
• High-dose volumes in mandible related to periodontal disease extractions.

## Introduction

Radiotherapy (RT) is an important treatment modality for head and neck cancer. It is associated with various oral side effects, such as dry mouth, dysphagia, dental problems, and osteoradionecrosis (ORN) [1]. When treating oropharyngeal cancer (OPSCC) with RT, the proximity of the tumour to the mandible is often associated with substantial exposure to intermediate-to-high radiation doses. This may trigger a pathophysiological process leading to ORN, a severe complication of RT in patients with head and neck cancer [2]. According to Wanifuchi et al. (2017), ORN is most frequently observed in the molar region, particularly in the mandible [3].

In addition to other risk factors, such as smoking and dental hygiene, several studies have established an association between post-RT tooth extractions and an increased lifetime risk of developing ORN [1], [2], [3], [4], [5], [6], [7], [8]. Therefore, patients with head and neck cancer undergo routine dental screenings prior to RT, in order to perform necessary pre-RT extractions to prevent post-RT complications [9].

Despite the implementation of preventive protocols and guidelines, post-RT extractions are frequently needed [8]. Currently, little is known about the incidence and baseline factors predictive of post-RT tooth extraction in a population undergoing RT. The purpose of this retrospective cohort study was to address this knowledge gap by reporting the incidence of post-RT tooth extraction and identifying patients at an increased risk of developing ORN. The clinical relevance of this study is that by enhancing our knowledge, we may be able to further personalise dental screening and prevention protocols to prevent post-RT tooth extraction and associated risk of ORN.

## Material and methods

### Patient cohort

**Fig. 1:**
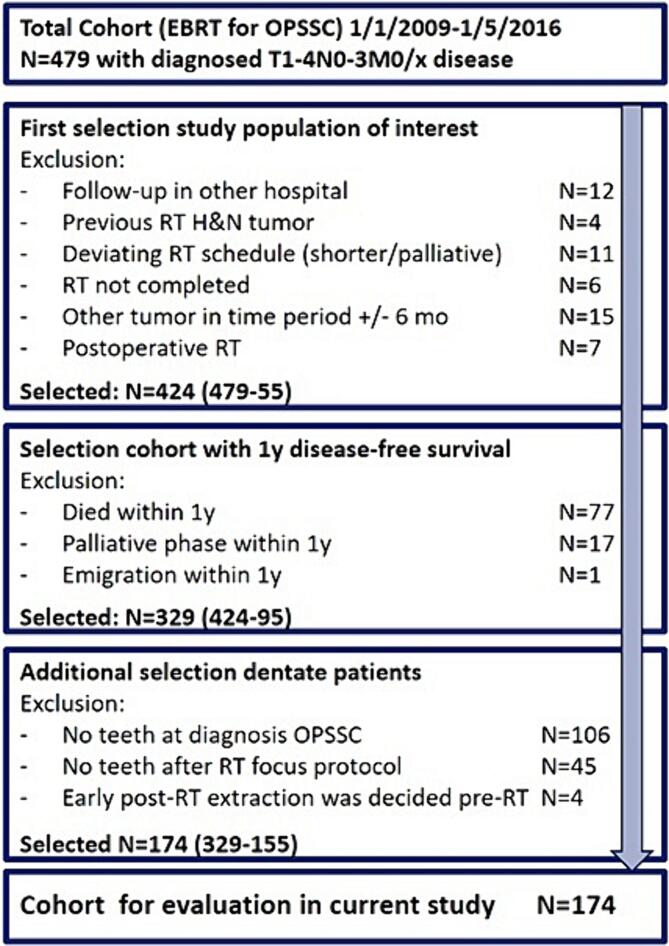
Flow diagram of patient selection. Abbreviations: EBRT = external beam radiotherapy, OPSCC = oropharyngeal squamous cell carcinoma.

The study protocol was reviewed and approved by the Erasmus MC Medical Ethics Review Committee (EMC17404). This retrospective cohort study reviewed patients diagnosed with OPSCC (T1-4, N0-3, M0) who received (chemo-) RT with curative intent at the Department of Radiotherapy of the Erasmus Medical Center between January 2009 and May 2016. For the current study, we included dentulous patients from this cohort with at least 1 year of follow-up record after the last RT session. Furthermore, we excluded patients who were diagnosed with other malignancies within an approximately 6-month window and those who had previously undergone RT in the head and neck region (more details in Fig. 1 and Table 1). This study complied with the strobe protocol.

**Table 1:** Baseline characteristics of the study population (N = 174).

**Characteristic**	**N patients (%)**
Age at the commencement of RT (mean + range, in years)	59.5; 32–86
Age categories, in years	
<60	87 (50 %)
60–70	69 (40 %)
>70	18 (10 %)
Sex	
Female	59 (34 %)
Male	115 (66 %)
WHO-performance score	
0	121 (70 %)
1	53 (30 %)
Smoker at diagnosis	
Current	68 (39 %)
Previous	71 (41 %)
Never	35 (20 %)
Oropharynx subsites	
Tonsil	87 (50 %)
Base of Tongue	54(31 %)
Soft Palate	11 (6 %)
Other	22 (13 %)
T-stage(clinical)	
T1-T2	115 (66 %)
T3-T4	59 (34 %)
Pre-radiation teeth extraction	
Yes (partly)	74 (43 %)
No	100 (57 %)
Fractions RT per week	
5 fractions	31 (18 %)
6 fractions	143 (82 %)
Chemoradiation	
Yes	59 (34 %)
No	115 (66 %)
RT of the neck	
One-sided	56 (32 %)
Left and right	118 (68 %)
RT-boost	
SBRT	108 (62 %)
IMRT	66 (38 %)
Oral hygiene as evaluated at baseline	
Poor	39 (22 %)
Borderline	17 (10 %)
Sufficient-Good	100 (58 %)
No data found	18 (10 %)

Abbreviations: WHO = World Health Organization, RT = radiotherapy, SBRT = stereotactic body radiotherapy, IMRT = intensity-modulated radiotherapy, SE = standard error.

### Treatment and follow-up

During weekly multidisciplinary meetings, the diagnoses and treatments of patients with OPSCC were assessed and discussed. Subsequently, the selected patients were treated with RT following standard clinical protocols. Each patient with head and neck cancer who was scheduled to undergo RT received pre-RT dental care. The assessment of oral hygiene occurred prior to radiation therapy and was conducted by a dental hygienist. The evaluation was based on plaque scores and periodontal conditions, specifically assessing bleeding and swelling. The resulting score categorized oral hygiene as either poor, borderline, or sufficient-good. If necessary, pre-RT dental extractions were performed according to the national guidelines. The current decision pattern outlined in this guidelines concerning removal of dental foci is based on empirical evidence and experience. Useful tools for this purpose include ‘dental risk factors’ and’malignancy-related risk factors’ [10], [11]. Based on the evaluation of dental and malignancy-related risk factors, the presence of strategic teeth, and overall clinical impression, a decision is made regarding which foci should be removed to prevent post-RT tooth extractions and the associated risk of ORN (Dijkstra et al. 2004).

Dental risk factors (Dijkstra et al. 2004):

• Periodontal inflammation
  o Pocket depth > 6 mm
  o Gingival recession > 6 mm
  o Spontaneous gingival bleeding
  o Furcation
  o Mobility > 2 mm
• Deep caries
• Root caries > 0.5 of the root circumference
• Periapical granulomas
• Internal and external root resorption
• Non-functional teeth
  o Partially impacted teeth
  o Residual tooth root
  o Fully impacted elements with follicular cyst
• Poor oral hygiene, often due to the patient's lack of motivation or cooperation

Malignancy-related risk factors (Dijkstra et al. 2004).

• Radiation dose > 55 Gy
• Molars in radiation field
• Teeth close to the tumour
• Time till radiotherapy < 14 days

Prior to RT, a thermoplastic mask was fitted to immobilise patients during RT. All patients received a total dose of 46 Gy delivered in 2 Gy fractions to the tumour location and indicated elective node regions in the neck area, followed by a boost to the specific tumour location and pathological lymph nodes. The boost was administered as a dose of 24 Gy delivered in 12 fractions of 2 Gy or 16.5 Gy delivered in 3 fractions of 5.5 Gy (a hypofractionated boost), which was only applicable in case of small tumour volumes. The intensity modulated radiotherapy (IMRT) was the selected technique of application. During treatment planning, effort was made to minimize radiation exposure to the organs at risk surrounding the target area. The mean dose to the contralateral parotid gland was maintained below 26 Gy when feasible, which allowed sparing of the parotid gland to preserve its salivary function [12]. Additional dose constraints for organs at risk within the oral cavity included mean dose of < 39 Gy and < 50 Gy to the submandibular glands and total oral cavity, respectively, whenever achievable.

Patients with large cT3-T4N0-2 M0 and cT1-4N3M0 tumours underwent a treatment regimen involving IMRT. The macroscopic tumour and pathological lymph nodes received a total dose of 70 Gy, delivered in five to six fractions of 2 Gy per week. In addition, elective lymph node regions were treated with a dose of 46 Gy using IMRT (referred to as the IMRT boost group).

For certain cases meeting specific criteria (cT3-T4 or N + and age ≤ 70), the treatment was further enhanced by including cisplatin or cetuximab. Cisplatin was administered at 100 mg/m^2^ on days 1, 22, and 43. Alternatively, cetuximab was administered at an initial dose of 400 mg/m^2^, followed by a weekly dose of 250 mg/m^2^.

In the context of IMRT, the target coverage objective was to achieve a high level of dose distribution. Specifically, the goal was to ensure that a minimum of 98 % of the planning target volume received at least 95 % of the prescribed dose. More details on the treatment can be found elsewhere [13], [14].

During RT, all patients regularly visited a dental hygienist and received weekly fluoride treatment using personalised teeth caps. Follow ups consisted of six visits in the first-year post RT. After the first year, follow ups decreased to a minimum of two visits per year for at least 5 years. Patient and their personal dentists were informed after the RT course that in case of indications for dental extractions, teeth had to be preferably extracted at the Oral and Maxillofacial surgery department of the Erasmus Medical Centre. Post-radiation extractions were conducted in accordance with clinical guidelines, with post-radiation tooth extraction mainly indicated when a tooth had a poor prognosis due to various reasons as listed in Table 2. When the radiotherapy dose in the region of the post-RT extraction had exceeded 40 Gy, a prophylactic strategy against osteoradionecrosis (ORN) was started. Antibiotic prophylaxis for extractions in an irradiated jaw included the protracted and heightened administration of amoxicillin (with clavulanic acid) at a dosage of 500 (+125) mg three times daily or clindamycin 600 mg three times daily. Antibiotics were started one day before treatment and continued for 10 days. Furthermore, it was suggested to patients to undergo hyperbaric oxygen therapy as an possible prophylactic strategy. Subsequent follow ups for these extractions was performed 2 weeks after extraction and 2–3 months after completion of RT via clinical examination and panoramic radiography.

**Table 2:** Details of post-RT tooth extractions within 7 year of follow-up (N = 49 patients).

**Variable**	**N (%)**
Main reason for extraction Radiation caries	18 (37 %)
- Chipped teeth	6 (12 %)
- Periodontal disease	22 (45 %)
Radiolucency / ORN	3 (6 %)
Extraction location(s)	
- Mandible	16 (33 %)
- Maxilla	16 (33 %)
- Both	17 (33 %)
Extracted teeth Incisors	6 (33 %)
- Canines	13 (27 %)
- Premolars	29 (58 %)
- Molars	37 (76 %)

Abbreviations: RT = radiotherapy, ORN = osteoradionecrosis.

### Data collection post-RT extractions and risk factors

Required data on post-RT extractions were collected on a separate case record form developed for the current study. This data collection took place without knowledge of the baseline data since these data were already collected earlier for this cohort. Medical records were retrospectively reviewed and radiographic images were compared obtained before and after RT (panoramic radiographs). Data included dates of extractions, the teeth extracted, locations, and indications for extraction. We evaluated patients’ data for up to 7 years of follow up, as follow-up data beyond this time point was not available for most patients. Data on the following were collected: radiation-induced caries, periodontal disease, periapical radiolucency, chipped teeth (excluding radiation-induced caries or periodontal disease or radiolucency), caries of a single element without indication of radiation caries, and other relevant factors. To determine the main reason for tooth extraction, the reasons provided in the medical correspondence were reviewed; if no clear reason was mentioned, patient medical records were reviewed to identify the main reason.

### Evaluated risk factors

The variables that were tested in this study were based on the existing literature regarding risk factors associated with dental problems and ORN. Notably, little literature is currently available concerning risk factors for post-RT tooth extraction [10], [11]. The evaluated factors included baseline clinical and dosimetric factors, as well as oral health status, as reported during the pre-RT visit for dental focus screening. The evaluated dose variables included mean dose delivered to the contralateral parotid gland, relative volume of the mandible receiving a dose > 60 Gy / 70 Gy / 80 Gy, and mean dose delivered to the mandible. All dose variables were calculated using an *α/β* ratio of 3 Gy to account for late effects.

### Statistical analyses

The time interval was calculated from the day of the final RT fraction. Follow up was concluded after 7 years (end of the evaluated follow up), at the time of death, entry into a palliative phase, diagnosis of a new primary tumour, or last hospital visit (whichever occurred first). Cox regression was used to evaluate potential risk factors for post-RT tooth extraction. For constructing the multivariable model, all risk factors with p < 0.2 in the univariable analysis were incorporated into a forward conditional selection procedure. The Kaplan–Meier method was used to calculate the cumulative incidences. Statistical significance was set at p < 0.05, and all statistical analyses were performed using the SPSS software (version 25, IBM Corporation, Armonk, NY, USA).

## Results

### Study population

Among the 479 patients diagnosed with OPSCC who were scheduled for RT at our department between January 2009 and May 2016, 424 completed RT with curative intent and were eligible according to our general set of exclusion criteria (Fig. 1). Within this group, we selected patients who had at least 1-year disease-free follow-up available and those who were dentate following RT, resulting in a final study group including 174 patients (Fig. 1). The median follow-up period was 4.9 years. Demographic and baseline characteristics of the study population are presented in Table 1. The mean age was 59.5 year; 66 % of the patients were men, and 43 % of them had undergone partial pre-RT extractions. Poor oral hygiene at baseline (at the visit for pre-RT dental screening) was noted in 23 % of the patients.

### Post-RT tooth extraction

**Fig. 2:**
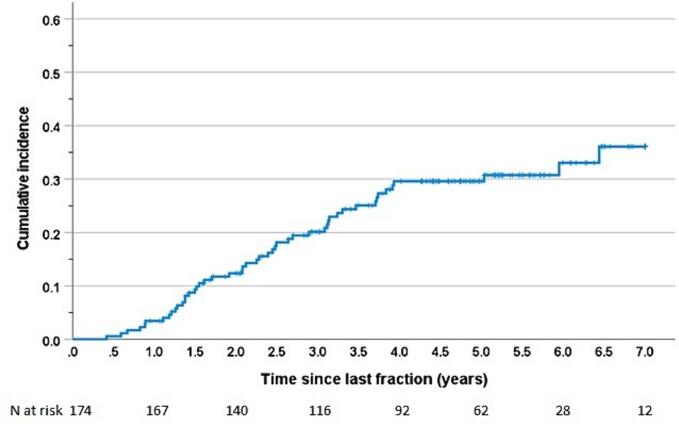
Cumulative incidence of the proportion of patients with post-radiotherapy tooth extraction (Kaplan–Meier estimate).

Among the 174 patients, 49 underwent post-RT tooth extraction, totalling 62 sessions (n = 37, one session; n = 11, two sessions; n = 1, three sessions). Characteristics of the extraction sites are listed in Table 2. Among these patients, 12 underwent extraction of one tooth, 16 underwent extraction of two or three teeth, 7 underwent extraction of four to nine teeth, and 14 underwent extraction of more than ten teeth, including 11 cases where the entire dentition was extracted. Most dental extractions were performed during the first 4 years of RT (Fig. 2). The estimated cumulative incidence of post-RT tooth extraction at 5 years was 30.7 % (3.8 % 1SE).

### Risk factors

In the univariable analysis, we identified several baseline risk factors significantly associated with an increased risk of post- RT tooth extraction (Table 3). These risk factors included alcohol abuse (hazard ratio [HR] = 2.27), poor oral hygiene (HR = 2.52), and relative volume of the mandible receiving a dose > 60 Gy (V60, HR = 1.36 per 10 % volume increase; V70, HR = 2.66 per 10 % volume increase; and V80, which indicated the presence of small volumes of very high dose hotspots, HR = 2.00 per 1 % volume increase).

**Table 3:** Univariable regression analysis of baseline factors for post-radiation teeth extraction.

	**Post-RT extraction** **(49 patients with event)**			**Extraction because of radiation caries** **(18 patients with event#)**			**Extraction because of periodontal bone loss** (24 patients with event*)		
**Variable**	**HR**	**95 % CI**	***p***	**HR**	**95 % CI**	***p***	**HR**	**95 % CI**	***p***
Sex (female vs. male)	1.25	0.7–2.2	0.5	1.07	0.4–2.9	0.9	1.31	0.6–3.0	0.5
Age (60 + vs. < 60)	1.34	0.8–2.4	0.3	1.00	0.4–2.5	1.00	1.42	0.6–3.2	0.4
Smoker (active vs. previous/none)	1.30	0.7–2.3	0.4	2.70	1.0–7.0	**0.040**	0.87	0.4–2.0	0.7
Alcohol abuse (history or current vs. no)	2.27	1.2–4.2	**0.008**	5.05	2.0–12.8	**<0.001**	1.76	0.7–4.4	0.2
Chemoradiation (yes vs. no)	1.34	0.8–2.4	0.3	1.94	0.8–4.9	0.16	1.16	0.5–2.6	0.7
Boost type (SBRT vs. standard)	0.95	0.5–1.7	0.9	0.60	0.2–1.5	0.3	1.47	0.6–3.5	0.4
Pre-RT extractions (yes vs. no)	0.76	0.4–1.4	0.4	1.05	0.4–2.7	0.9	0.53	0.2–1.3	0.16
Oral hygiene (poor vs. other)	2.52	1.4–4.5	**0.002**	4.34	1.7–11.0	**0.002**	1.44	0.6–3.6	0.4
Mean dose spared parotid gland (per 10 Gy increase)	1.26	1.0–1.6	0.087	1.85	1.2–2.8	**0.005**	1.00	0.7–1.5	1.0
Mean dose mandible (per 10 Gy)	1.30	0.9–1.9	0.16	2.13	1.20–3.8	**0.011**	0.93	0.5–1.8	0.8
% Mandible dose > 60 Gy (per 10 %)	1.36	1.1–1.7	**0.014**	1.93	1.4–2.7	**<0.001**	1.08	0.7–1.7	0.8
% Mandible dose > 70 Gy (per 10 %)	2.66	1.5–4.7	**<0.001**	6.18	2.9–13.4	**<0.001**	2.40	0.9–6.7	0.097
% Mandible dose > 80 Gy (per 1 %)	2.00	1.4–2.9	**<0.001**	2.06	1.1–3.7	**0.017**	2.55	1.5–4.2	**<0.001**
Mandible volume (<vs. > median volume)	0.88	0.5–1.6	0.7	0.64	0.2–1.8	0.4	0.64	0.2–1.8	0.4

# For all patients with radiation caries, elements in the mandible were removed * Only extractions in the mandible were used for analysis of mandibular dose variables (n = 16 events).

Abbreviations: HR=Hazard Ratio, RT=radiotherapy, SBRT=stereotactic boost radiotherapy, 95% CI=95% confidence interval.

When evaluating post-RT extractions related specifically to radiation caries, we identified the following risk factors: active smoking (HR = 2.95), alcohol abuse (HR = 4.60), poor oral hygiene (HR = 3.95), mean dose to the contralateral spared parotid gland (HR = 1.85 per 10 Gy), and all evaluated dose parameters of the mandible (highest HR of 6.18 observed for V70) (Table 3). For post-RT extractions associated with periodontal disease, we observed a correlation with the hotspot variable V80 when evaluating extractions that included the mandible. This correlation was observed in 16 of the 24 patients who underwent extraction owing to periodontal disease.

In multivariable analysis focusing on the endpoint of radiation caries, the following factors remained significant in the model (Table 4): V70 (HR = 8.30), active smoking (HR = 2.33), and poor oral hygiene (HR = 5.49).

**Table 4:** Results of multivariable regression analysis for the endpoint of post-radiation teeth extraction indicated by radiation caries (N = 18 events).

**Variables**	**HR**	**95 % CI**	***p***
Mandible V70 per 10 %	8.30	3.4–20.2	<0.001
Active smoker yes vs. no	2.33	1.1–5.0	0.032
Oral Hygiene poor vs. other	5.49	2.0–14.8	<0.001

Abbreviations: HR=Hazard Ratio, 95% CI= 95% confidence interval, V70=volume receiving ≥70 Gy.

### Osteoradionecrosis

We observed 26 cases of ORN for which follow-up data were available (n = 24, mandibles; n = 2, maxilla). Among them, 14 patients developed ORN specifically after pre-RT extractions, of whom 6 also underwent post-RT extractions later (in two patients, the post-RT extractions were related to the previously developed ORN). Seven patients developed ORN after post-RT extraction, and two of them underwent both pre- and post-RT extractions. Five patients developed ORN with no previous history of pre- or post-RT extractions, and four of these patients later underwent post-RT extractions (in one patient, post-RT extraction was mainly related to ORN).

## Discussion

In the present retrospective cohort study, we evaluated the incidence and risk factors for post-RT extractions in dentate patients diagnosed with OPSCC who had previously undergone curative-intent RT. We observed a cumulative incidence of 31 % at 5 years of follow up.

Exclusively, the OPSCC group was intentionally chosen to ensure homogeneity within the study population. Significant divergences in treatment modalities and chronological sequences exist between OPSCC and oral cavity cancer. This methodological selection aims to enhance internal validity.The primary reasons for extraction were radiation caries and periodontal disease. The personalised risk profile of patients who had undergone post-RT extractions related to radiation caries differed considerably from the risk profile for extractions related to periodontal disease. For radiation caries, we identified multiple clinical and dosimetric variables, whereas for periodontal disease, only the presence of hotspots in the mandible correlated with this endpoint. To our knowledge, this is the first study to investigate the risk factors for post-RT tooth extraction classified by the cause of dental failure.

The prognostic value of small high-dose volumes for dental late toxicity has been previously reported in this study cohort regarding osteoradionecrosis (ORN). Baker et al and Verduijn et al reported that these high-dose volumes, particularly present in patients treated with stereotactic hypofractionated boost (SBRT), were predictive for ORN [13], [15]. In our current study, we also find this variable to be prognostic for post-RT extractions. However, the variable SBRT itself did not emerge as a significant univariable predictive factor. This can be partially attributed to the fact that selected SBRT patients are treated for small tumours, in contrast to non-SBRT patients who do not meet the criteria for small tumour eligibility for SBRT. Verduijn et al similarly found SBRT not to be a univariable predictive factor for ORN due to this phenomenon. Therefore, we conclude that high-dose volumes associated with SBRT increase the risks of late dental toxicities in a selectively treated patient population with relatively small tumour volumes and relatively low baseline risks for late dental toxicities.

The national guidelines recommend pre-RT oral assessment and associated extractions based on dental and malignancy-related risk factors to minimise the likelihood of post-RT tooth extraction, thereby preventing the risk of developing ORN and other associated complications (Dijkstra et al. 2004). In our study, approximately one-third of the dentulous patients required post-RT teeth extraction which was not initially considered at risk before RT. The findings of this study, along with current guidelines, could be valuable to inform patients about the increased risk of post-RT extraction at an early stage, support them with specific precautionary measures, and aid in making decisions regarding pre-RT tooth extraction for individual cases. In our cohort, seven patients developed ORN after post-RT extraction, suggesting that incorporating personalised and patient-specific risk factors into the current dental screening protocols might have prevented these cases.

Post-RT tooth extraction was primarily indicated for periodontal disease and radiation-induced caries. We observed correlations between risk factors, including active smoking, alcohol abuse, poor oral hygiene, a higher mandibular dose, a higher mean dose to the contralateral spared parotid gland, and an increased risk of extractions caused by radiation caries. Recently, Brennan et al. (2022) reported the incidence and risk factors of tooth failure (with 24 months of follow-up) in a prospective cohort study including 572 patients with head and neck cancer [16]. They reported a cumulative incidence of 18 %, which closely aligns with 17 % observed in our study at 2 years. They identified various associated risk factors, including a low number of teeth at baseline, older age, active caries at baseline, proton therapy as a preventive factor, RT dose, oral hygiene compliance, and salivary flow. Notably, they did not specify the different types of tooth failure as we did in our study, nor did they examine specific dose levels in the parotid glands and mandible. A relationship between the mean dose to the spared parotid gland and radiation caries has been previously reported in patients with head and neck cancer patients by Hey et al. and Gomez et al. [17], [18]. In agreement with these results, we observed a significant relationship between the mean dose to the spared parotid gland and risk of post-RT extraction due to radiation-induced caries. Gomez et al. also reported that high-dose levels in the mandible are associated with post-RT dental extractions [17]. A relationship between alcohol abuse and periodontal disease has been reported in literature [19]; however, no specific association has been observed for radiation-induced caries, as observed in our study. In our study, alcohol abuse was identified a significant factor in the univariable analysis for post-RT extraction due to radiation-induced caries; however, it was not included in the multivariable model, possibly because of its high correlation with active smoking and poor oral hygiene.

Patients who receive RT in the head and neck area are at an increased risk of developing periodontal disease compared to the general population^1^. RT causes hyposalivation and changes in oral microbiome, which may lead to a loss of salivary protection, leading to periodontal disease [1], [10]. In our study, we observed that the molars were the teeth most frequently extracted after RT, with periodontal decay being the main reason for their extraction. This location also carries a greater risk of developing ORN [1], [3].

In this study, standard follow up at the outpatient clinic was concluded around the fifth year if a patient had no tumour relapse and experienced no side effects requiring further attention. However, in the event of dental problems, the patient was strongly advised to return to the outpatient clinic even after 5 years. Therefore, the estimated cumulative incidences beyond 5 years might be somewhat biased because only patients experiencing problems continued their follow-up after the 5-year oncologic follow up.

This study had several limitations. All the data were collected retrospectively; therefore, there is a possibility that post-RT extractions were performed outside the Erasmus Medical Centre and were not reported in the electronic patient records, despite strong recommendations for patients and their dentists to refer the patient back to the Erasmus Medical Centre in case of tooth failure. During the clinical follow-up of 5 years, such cases were typically identified because patients or their dentists reported this and we could score this information in our database. Therefore, we expect that possible underestimations of the incidence of post-RT extractions up to 5 years will be very limited, and beyond 5 years of follow-up we expect that cases were indeed missed. We expect that this has not introduced significant attrition bias to the estimated hazard ratios. Additionally, as inclusion criteria, we adopted a minimum 1-year tumour-free survival, leading to the exclusion of many T3 and T4 cases. This resulted in an imbalance in certain patient characteristics. Based on the extensiveness of medical records prior to treatment, we can assume that the baseline variables and risk factors that we identified were reliable. However, we were unable to collect reliable data on compliance with oral hygiene instructions, use of fluoride caps, and visits to oral hygienists, which are regarded as relevant factors for preventing tooth failure following RT. In conclusion, dentulous patients with OPSCC have a considerable risk of post-RT extractions. Identified risk factors in this study included poor oral hygiene, active smoking, parotid gland dose, and high-dose volumes in the mandible. Although the national guidelines focus on the elimination of dental foci prior to RT to prevent post- RT tooth extraction, our findings indicated that approximately one-third of patients require post- RT tooth extraction. To better serve the main goal of ORN prevention, we suggest incorporating information concerning these personalised risk factors for post-RT tooth extraction to further minimise the risks associated with RT and improve patient outcomes.

## Contribution author(s)

Study concepts: D. Chin, W.D. Heemsbergen, E.B. Wolvius, G.M. Verduijn, H. Mast, B.P. Jonker.

Study design: W.D. Heemsbergen, E.B. Wolvius, G.M. Verduijn, S.F. Petit, H. Mast, F.R. Rozema.

Data acquisition: D. Chin, W.D. Heemsbergen, G. Verduijn, H. Mast.

Quality control of data and algorithms: D. Chin, W.D. Heemsbergen, H. Mast.

Data analysis and interpretation: D. Chin, W.D. Heemsbergen, E.B. Wolvius, G.M. Verduijn, S.F. Petit, H. Mast, F.R. Rozema, B.P. Jonker, M.M. Möring.

Statistical analysis: D. Chin, W.D. heemsbergen.

Manuscript preparation: D. Chin, W.D. Heemsbergen, B.P. Jonker.

Manuscript editing: D. Chin, W.D. Heemsbergen, E.B. Wolvius, G.M. Verduijn, S.F. Petit, H. Mast, F.R. Rozema, B.P. Jonker, M.M. Möring.

Manuscript review: D. Chin, W.D. Heemsbergen, E.B. Wolvius, G.M. Verduijn, S.F. Petit, H. Mast, F.R. Rozema, B.P. Jonker, M.M. Möring.

All authors gave their final approval and agree to be accountable for all aspects of the work.

## Conflicts of interest statement

The authors declare no conflict of interest.

## Data availability statement

This study data are available upon reasonable request.

## Ethics statement

This study was reviewed by the Erasmus MC Medical ethics Review Committee (nr EMC17404) and permission was obtained to conduct the study adhering local, national and international guidelines.

## Funding acknowledgement

This research received no specific grant from any funding agency in the public, commercial, or not-for-profit sectors.

## CRediT authorship contribution statement

**Denzel Chin:** Conceptualization, Methodology, Software, Validation, Formal analysis, Investigation, Resources, Data Curation, Writing – Original Draft, Writing – Review & Editing, Visualization. Project administration. **Hetty Mast:** Conceptualization, Data Curation, Writing – Review & Editing, Visualization. **Gerda M. Verduijn:** Conceptualization, Writing – Review & Editing, Visualization. **Michelle Möring:** Writing – Review & Editing. **Steven F. Petit:** Writing – Review & Editing, Visualization. **Frederik R. Rozema:** Writing – Review & Editing, Visualization. **Eppo B. Wolvius:** Conceptualization, Writing – Review & Editing, Visualization. **Brend P. Jonker:** Conceptualization, Writing – Review & Editing, Visualization. **W.D. Heemsbergen:** Conceptualization, Methodology, Software, Validation, Formal analysis, Investigation, Resources, Data Curation, Writing - Original Draft, Writing – Review & Editing, Visualization, Supervision, Project administration.

## Declaration of competing interest

The authors declare that they have no known competing financial interests or personal relationships that could have appeared to influence the work reported in this paper.
