# Supplementary material for: Personalizing dental screening and prevention protocols in dentulous patients with oropharyngeal cancer undergoing radiotherapy: A retrospective cohort study
Source: Clin Transl Radiat Oncol. 2024 Mar 7;46:100759. doi: 10.1016/j.ctro.2024.100759 (PMC10940125; doi:10.1016/j.ctro.2024.100759)
Supplement: Supplementary data 1 [file mmc1.docx]

STROBE Statement—checklist of items that should be included in reports of observational studies

|  | Item No | Recommendation |
| --- | --- | --- |
| **Title and abstract** | 1 | (*a*) Indicate the study’s design with a commonly used term in the title or the abstract  Retroscpective cohort study as stated in the title on page 1 and Material and Methods on page 4. |
|  |  | (*b*) Provide in the abstract an informative and balanced summary of what was done and what was found  Provided on page 2. |
| Introduction | | |
| Background/rationale | 2 | Explain the scientific background and rationale for the investigation being reported  Included in the Introduction on page 3. |
| Objectives | 3 | State specific objectives, including any prespecified hypotheses  Included in the Introduction on page 3. |
| Methods | | |
| Study design | 4 | Present key elements of study design early in the paper  Included in the Material and Methods on page 4. |
| Setting | 5 | Describe the setting, locations, and relevant dates, including periods of recruitment, exposure, follow-up, and data collection  Included in the Material and Methods on pages 4,5,6 and 7. |
| Participants | 6 | (*a*) *Cohort study*—Give the eligibility criteria, and the sources and methods of selection of participants. Describe methods of follow-up  *Case-control study*—Give the eligibility criteria, and the sources and methods of case ascertainment and control selection. Give the rationale for the choice of cases and controls  *Cross-sectional study*—Give the eligibility criteria, and the sources and methods of selection of participants  Included in the Material and Methods on pages 4,5,6 and 7. |
|  |  | (*b*) *Cohort study*—For matched studies, give matching criteria and number of exposed and unexposed  *Case-control study*—For matched studies, give matching criteria and the number of controls per case  Not applicable. |
| Variables | 7 | Clearly define all outcomes, exposures, predictors, potential confounders, and effect modifiers. Give diagnostic criteria, if applicable  Included in the Material and Methods on pages 4,5,6 and 7. |
| Data sources/ measurement | 8* | For each variable of interest, give sources of data and details of methods of assessment (measurement). Describe comparability of assessment methods if there is more than one group.  Included in the Material and Methods on pages 4,5,6 and 7. |
| Bias | 9 | Describe any efforts to address potential sources of bias  Addressed in the discussion on page 13. |
| Study size | 10 | Explain how the study size was arrived at  Included in the Material and Methods on page 4 (an d figure 1) |
| Quantitative variables | 11 | Explain how quantitative variables were handled in the analyses. If applicable, describe which groupings were chosen and why  Included in the Material and Methods on pages 8 and 9. |
| Statistical methods | 12 | (*a*) Describe all statistical methods, including those used to control for confounding  Included in the Material and Methods on page 9. |
|  |  | (*b*) Describe any methods used to examine subgroups and interactions  Included in the Material and Methods on page 9. |
|  |  | (*c*) Explain how missing data were addressed  Included in the Material and Methods on page 8. |
|  |  | (*d*) *Cohort study*—If applicable, explain how loss to follow-up was addressed  *Case-control study*—If applicable, explain how matching of cases and controls was addressed  *Cross-sectional study*—If applicable, describe analytical methods taking account of sampling strategy  Included in the Material and Methods on page 8. |
|  |  | (*e*) Describe any sensitivity analyses  Not applicable. |

Continued on next page

| Results | | |
| --- | --- | --- |
| Participants | 13* | 1. Report numbers of individuals at each stage of study—eg numbers potentially eligible, examined for eligibility, confirmed eligible, included in the study, completing follow-up, and analysed   Included in the results on page 10. |
|  |  | 1. Give reasons for non-participation at each stage   Included in the results on page 10 (and figure 1) |
|  |  | 1. Consider use of a flow diagram   Figure 1. |
| Descriptive data | 14* | (a) Give characteristics of study participants (eg demographic, clinical, social) and information on exposures and potential confounders  Included in the results on page 10 and Table 1. |
|  |  | (b) Indicate number of participants with missing data for each variable of interest  Included in the results on page 10. |
|  |  | (c) *Cohort study*—Summarise follow-up time (eg, average and total amount)  Included in the results on page 10. |
| Outcome data | 15* | *Cohort study*—Report numbers of outcome events or summary measures over time |
|  |  | *Case-control study—*Report numbers in each exposure category, or summary measures of exposure |
|  |  | *Cross-sectional study—*Report numbers of outcome events or summary measures  Included in the results on page 10 and figure 2 and table 2. |
| Main results | 16 | (*a*) Give unadjusted estimates and, if applicable, confounder-adjusted estimates and their precision (eg, 95% confidence interval). Make clear which confounders were adjusted for and why they were included |
|  |  | (*b*) Report category boundaries when continuous variables were categorized |
|  |  | (*c*) If relevant, consider translating estimates of relative risk into absolute risk for a meaningful time period  Included in the results on page 11 and table 3. |
| Other analyses | 17 | Report other analyses done—eg analyses of subgroups and interactions, and sensitivity analyses  Not applicable. |
| Discussion | | |
| Key results | 18 | Summarise key results with reference to study objectives  Included in the discussion on page 12. |
| Limitations | 19 | Discuss limitations of the study, taking into account sources of potential bias or imprecision. Discuss both direction and magnitude of any potential bias  Included in the discussion on page 14. |
| Interpretation | 20 | Give a cautious overall interpretation of results considering objectives, limitations, multiplicity of analyses, results from similar studies, and other relevant evidence  Included in the discussion on page 13. |
| Generalisability | 21 | Discuss the generalisability (external validity) of the study results  Included in the discussion on page 14. |
| Other information | | |
| Funding | 22 | Give the source of funding and the role of the funders for the present study and, if applicable, for the original study on which the present article is based  Not applicable. |

*Give information separately for cases and controls in case-control studies and, if applicable, for exposed and unexposed groups in cohort and cross-sectional studies.

**Note:** An Explanation and Elaboration article discusses each checklist item and gives methodological background and published examples of transparent reporting. The STROBE checklist is best used in conjunction with this article (freely available on the Web sites of PLoS Medicine at http://www.plosmedicine.org/, Annals of Internal Medicine at http://www.annals.org/, and Epidemiology at http://www.epidem.com/). Information on the STROBE Initiative is available at www.strobe-statement.org.
