# Supplementary material for: Personalizing dental screening and prevention protocols in dentulous patients with oropharyngeal cancer undergoing radiotherapy: A retrospective cohort study
Source: Clin Transl Radiat Oncol. 2024 Mar 7;46:100759. doi: 10.1016/j.ctro.2024.100759 (PMC10940125; doi:10.1016/j.ctro.2024.100759)
Supplement: Supplementary data 2 [file mmc2.pdf]

Wiley Editing Services

ENGLISH EDITING CERTIFICATE

This document certifies that the manuscript listed below was edited for proper English language, grammar, punctuation, spelling, and overall style by one or more of the highly qualified native English speaking editors at Wiley Editing Services

Manuscript title

Personalizing dental screening and prevention protocols in dentulous patients with oropharyngeal cancer undergoing radiotherapy: a retrospective cohort study

Authors

Denzel Chin, Hetty Mast, Gerda Verduijn, Michelle Möring, Steven Petit, Frederik Rozema, Eppo Wolvius, Brend Jonker, Wilma Heemsbergen

Order No

CLVTI_1

Date Issued

September 18, 2023

This document certifies that the manuscript listed above was edited for proper English language, grammar, punctuation, spelling, and overall style. Neither the research content nor the authors' intentions were altered in any way during the editing process. Documents receiving this certification should be English-ready for publication; however, the author has the ability to accept or reject our suggestions and changes. If you have any questions or concerns about this document or certification, please contact help@wileyeditingservices.com.

Wiley Publishing Services is a service of Wiley Publishing. Wiley's Scientific, Technical, Medical, and Scholarly (STMS) business serves the world's research and scholarly communities, and is the largest publisher for professional and scholarly societies. Wiley is committed to providing high quality services for researchers. To find out more about Wiley Editing Services, visit

WILEY

http://wileyeditingservices.com. To learn more about our other author services provided by Wiley Publishing, visit https://authorservices.wiley.com/
